# Does an “EZ” Survey Improve the Data Quality of the Consumer Assessment of Healthcare Providers and Systems (CAHPS^®^) Clinician and Group Survey 3.1?

**DOI:** 10.1177/23743735241297622

**Published:** 2024-11-14

**Authors:** Ron D. Hays, Julie A. Brown, Charleen Mikail, Denise D. Quigley

**Affiliations:** 1 Department of Medicine, UCLA Division of General Internal Medicine & Health Services Research, Los Angeles, CA, USA; 2 1312 RAND Corporation; Santa Monica, CA, USA; 3 ChapCare by AltaMed Health Services Corporation; Los Angeles, CA, USA

**Keywords:** patient experience, survey data, quantitative methods, outpatient care data

## Abstract

Completing self-administered patient experience surveys is challenging for many patients. We randomized adult patients receiving care from an urban safety net provider to complete the Consumer Assessment of Healthcare Providers and Systems (CAHPS^®^) Clinician and Group Survey 3.1 (CG-CAHPS 3.1), or an “EZ” survey created using plain language principles. We compared response rates, item missingness, item-scale correlations, and reliability of patient experience scores based on 264 completed surveys (64% female, 66% Hispanic, 33% high school education or less). The CG-CAHPS 3.1 survey response rate was higher (20% vs 16%), and failure to follow skip instructions was more common for the EZ survey. Internal consistency reliability for multi-item scales was similar, but provider-level reliability was higher for the EZ than for the CG-CAHPS 3.1 survey measures. Cognitive interviews with patients are needed to assess whether the wording of the EZ survey is responsible for the lower response rates and more skip pattern errors. Future studies are also required to provide additional information about the psychometric properties of the CG-CAHPS 3.1 and EZ surveys.

## Key Findings

1. The Consumer Assessment of Healthcare Providers and Systems (CAHPS^®^) Clinician and Group Survey 3.1 (CG-CAHPS 3.1) yielded a higher response rate than an EZ survey.
2. The CG-CAHPS 3.1 survey produced fewer skip pattern errors by respondents than the EZ survey.
3. The provider-level reliability of EZ survey measures was higher than that of the CG-CAHPS 3.1 survey.
4. The response rate for those offered a $2 versus a $5 incentive did not differ.

## Introduction

The Consumer Assessment of Healthcare Providers and Systems (CAHPS^®^) suite of surveys includes items to evaluate experiences with health plans (eg, health plan and home and community-based services), providers (eg, clinician, medical group, hospice, home health, and surgical care), facility-based care (hospital, in-center hemodialysis, nursing home, and outpatient ambulatory surgery center), and condition-specific care (cancer and mental health). CAHPS survey data are used as a component of provider quality payments, including pay-for reporting, hospital value-based purchasing payments, quality bonus payments for Medicare Advantage Plans, and dialysis center value-based purchasing payments.^1^ CAHPS survey users include patients, families, caregivers, healthcare purchasers, healthcare accreditation organizations, providers, health plans, and improvement collaboratives.^2,3^

Surveys must be accessible to ensure that care experiences reflect a representative sample of the target population. Individuals from underserved groups have been shown to have some difficulty completing CAHPS surveys.^4^ Survey completion barriers must be addressed to ensure generalizable research results.^5^

The CAHPS Clinician and Group Survey 3.1 (CG-CAHPS 3.1) was intended to be readable for those with a 7th-grade education or higher. However, the Flesch-Kincaid reading level estimates^6,7^ exceed the target level for 10 of the 12 CG-CAHPS 3.1 core items (Table 1). Supplemental Figure 1 shows an example of a CG-CAHPS 3.1 item estimated to require more than a high school education. The example item has several words written in the passive voice, and line length determines wrapping to the following line. An alternative to this item was created using methods designed to improve the readability^8^ and comprehensibility of questions in healthcare surveys.^9^

**Table 1.** CG-CAHPS 3.1 Survey and EZ Survey Items (Flesch-Kincaid Reading Level).

CG-CAHPS 3.1 Survey	EZ Survey
*Timely Care*	*Timely Care*
6. In the past 6 months, when you contacted this provider's office to get an appointment for care you needed right away, how often did you get an appointment as soon as you needed? (13.7)	6. How often did you get *care as soon as you needed*? (2.6)
8. In the past 6 months, when you made an appointment for a check-up or routine care with this provider, how often did you get an appointment as soon as you needed? (12.1)	8. How often did you get an appointment *as soon as you needed*? (4.8)
10. In the past 6 months, when you contacted this provider's office during regular office hours, how often did you get an answer to your medical question that same day? (13.2)	10. How often did you get answers to your medical questions *the same day*? (5.8)
*Communication*	*Communication*
11. In the past 6 months, how often did this provider explain things in a way that was easy to understand? (8.1)	13. How often did this doctor *explain things* in a way you understood? (5.8)
12. In the past 6 months, how often did this provider listen carefully to you? (7.5)	14. How often did this doctor *listen to you carefully*? (7.5)
14. In the past 6 months, how often did this provider show respect for what you had to say? (5.8)	16. How often did this doctor *show respect* for what you had to say? (4.0)
15. In the past 6 months, how often did this provider spend enough time with you? (5.2)	17. How often did this doctor *spend enough time* with you? (3.6)
*Coordination of care*	*Coordination of care*
13. In the past 6 months, how often did this provider seem to know the important information about your medical history? (11.6)	15. How often did this doctor *seem to know* what is important to you about your health? (6.1)
17. In the past 6 months, when this provider ordered a blood test, X-ray, or other test for you, how often did someone from this provider's office follow up to give you those results? (13.3)	19. How often did this doctor *explain the test results to you*? (4.7)
20. In the past 6 months, how often did you and someone from this provider's office talk about all the prescription medicines you were taking? (11.4)	21. How often did this doctor *talk about all the medicine* you took? (5.8)
*Office staff*	*Office staff*
21. In the past 6 months, how often were clerks and receptionists at this provider's office as helpful as you thought they should be? (9.2)	11. How often were clerks and receptionists as *helpful as they should be* in the past 6 months? (6.3)
22. In the past 6 months, how often did clerks and receptionists at this provider's office treat you with courtesy and respect? (10.0)	12. How often did clerks and receptionists treat you with *respect* in the past 6 months? (5.9)

Abbreviations: CAHPS^®^, Consumer Assessment of Healthcare Providers and Systems; CG-CAHPS 3.1, Clinician and Group Survey 3.1.

We produced an EZ version of the CG-CAHPS 3.1 survey using plain language principles: *grammatic parsing* and *stanzaic versification* (Table 1). Grammatic parsing identifies conjunctives (connector words within sentences) and parses compound and complex sentences into their grammatical components (phrases and clauses); stanzaic versification converts simple survey sentences into 2 or 3 shorter lines to create a survey akin to a stanza in poetry. Each line of the survey stanza, usually a phrase or clause, represents one idea. Unlike the unsystematic truncation produced by automatic text wrapping from word processing programs, stanzaic versification breaks are designed to represent a single idea. The alternative “EZ” item (shown in Supplemental Figure 1) is short, presents a single thought on each line, and reduces the estimated reading level to 3rd grade.

The simplification process included changes that could alter the meaning of the corresponding items. In particular, the CG-CAHPS 3.1 items about timely care and office staff refer to this provider's office, but the EZ items do not. The CG-CAHPS 3.1 communication and coordination of care items refer to “provider,” whereas the EZ survey refers to “doctor.” Finally, a CG-CAHPS 3.1 survey item refers to prescription medicines, but the corresponding EZ item relates to medicine.

The study aimed to inform possible modifications to patient experience surveys. We compare response rates, item missingness, item distributions, item-scale correlations, and reliability of patient experience scores for the CG-CAHPS 3.1 and EZ surveys.

## Method

### CG-CAHPS 3.1 Survey

The 31-item CG-CAHPS 3.1 survey includes 9 “About You” questions. It also has one global rating question: “Using any number from 0 to 10, where 0 is the worst provider possible, what number would you use to rate this provider?” Twelve questions were used to elicit patient reports about healthcare:

### EZ Survey

The EZ survey included 31 questions, including 9 “About You” questions. One global rating question was included: “Rate the care this doctor gave you in the last 6 months. Pick a number from 0 to 10. The Worst doctor is 0. The Best doctor is 10.” Twelve questions were used to elicit patient reports about healthcare.

## Sampling

We obtained a list of 3167 patients who had visited a provider in the past 6 months at 1 of 17 Federally Qualified Health Center urban clinics in Los Angeles. We drew a random sample of 1600 patients and randomized 800 to complete the CG-CAHPS 3.1 survey and the other 800 to complete the EZ survey. We followed CAHPS guidelines and did not sample more than 1 person per household. To see if differing incentives improved response rates, we randomized patients to 1 of 2 incentive amounts: $2 and $5. Incentives were postpaid, contingent upon returning a survey. The visit dates used for sampling ranged from January 16, 2023 through April 14, 2023, and survey completion dates ranged from August 1, 2023 through December 21, 2023.

Several aspects of our field test procedures associated with increasing responses to mail surveys were implemented^10,11^:

- Mailing a prenotification letter in advance of the survey with the logo of the healthcare organization,
- Using first-class postage,
- Sending a second survey if there is no response to the first mailing,
- Providing a Spanish language survey to those with a Spanish language preference noted in their medical record, and
- Offering an incentive for completing the survey.

## Analysis

First, we compare response rates to the CG-CAHPS 3.1 and EZ surveys. Then, we summarize rates of failure to follow skip patterns. We calculated rates of item missingness, item frequencies and means, standard deviations (SDs), and estimated alpha internal consistency reliability coefficients^12^ for the timely care, office staff, communication, and care coordination composites. We estimated the means and SDs for the global rating of the doctor item. We computed product-moment correlations among the composites and the global rating item at the patient level. We estimated doctor-level reliability and doctor-level correlations among the patient experience measures. We also estimated item-scale correlations^13^ and examined indicators of the number of underlying factors: Guttman's^14^ weakest lower bound and the scree test.^15^ Then, we evaluated a categorical confirmatory factor analysis model based on the comparative fit index^16^ and the root mean square error of approximation.^17^

All analyses were conducted using SAS 9.4 (TS Level 1M7) software.

## Results

### Sample

There were no significant differences between the randomized groups in the number of mailings (EZ mean = 2.01 vs CG-CAHPS 3.1 mean = 2.00, t = 0.47, *P* = .6418), preferred language (EZ 40% Spanish vs CG-CAHPS 3.1 38% Spanish, χ^2^ = 0.62, df = 1, *P* = .4311), or type of provider on the sampled visit (χ^2^ = 2.89, df = 2, *P* = .2360).

We received 264 completed surveys (147 CG-CAHPS 3.1 survey and 117 EZ survey), a mean of 198 days after the sampled visit (range of 130-329 days) for a response rate of 18% after excluding the 104 surveys that were ineligible due to being undeliverable. The CG-CAHPS 3.1 survey response rate was significantly higher than the EZ survey (20% vs 16%; χ^2^ = 3.85, df = 1, *P* = .0497). Response rates did not differ significantly by $5 versus $2 incentive: EZ survey (t = 0.81, df = 743, *P* = .4201) and CG-CAHPS 3.1 survey (t = 0.36, df = 749, *P* = .7177).

Most of the sample was female (64%). In addition, 66% were Hispanic, 14% Black, 14% White, and 7% Asian. Thirty-three percent reported a high school education or less. Forty-four percent of the surveys were completed in Spanish. The modal age category was 55 to 64 (40%), and 77% of the sample was between 45 and 74 years old. Self-rated physical health was 3% *poor*, 27% *fair*, 40% *good*, 15% *very good*, and 15% *excellent*. Self-rated mental health was 3% *poor*, 19% *fair*, 36% *good*, 18% *very good*, and 24% *excellent*.

Thirteen CG-CAHPS 3.1 and 18 EZ survey respondents reported not getting care from the named doctor in the past 6 months, so the analytic sample was 232 surveys (n = 133 CG-CAHPS 3.1 survey and n = 99 EZ survey). The patient sample provided assessments of 16 healthcare providers: 7 physicians, 5 nurse practitioners, and 5 physician assistants.

Among survey respondents, there were no significant differences between CG-CAHPS 3.1 and EZ groups on the $2 versus $5 incentive (t = 0.76, *P* = .4488), number of mailings (CG-CAHPS 3.1 mean = 1.51, EZ mean = 1.46, t = 0.56, *P* = .5760), days between sampled visit and survey return (CG-CAHPS 3.1 mean = 202, EZ mean = 193, t = 1.73, *P* = .0844), and preferred language (CG 3.1 Spanish = 40%, EZ Spanish = 45%, χ2 = 0.79, df = 1, *P* = .3739). However, there was a significant difference between CG-CAHPS 3.1 and EZ groups on the wave when the survey was returned (CG 3.1 mean = 1.32, EZ mean = 1.10, t = 3.47, *P* = .0006).

### Failure to Skip

The Supplemental Appendix details the percentage of the eligible sample failing to follow each of the 8 skip pattern sequences. The overall rate of failing to skip was significantly higher for the EZ (26%) than for the CG-CAHPS 3.1 survey (16%): χ^2^ = 8.04, df = 1, *P* = .0046 (95% confidence interval around the difference 3% to 17%).

### Item Missing Data Rates

The average number of items missing for the 24 items asked of everyone was small and not significantly different between the EZ and CG-CAHPS 3.1 surveys (1.04 vs 0.53, t = 1.78, df = 1, *P* = .0769).

### Item Frequencies

We forward cleaned data based on screener responses so that questions following a screener that were supposed to be skipped were recoded to missing data. Supplemental Table 1 provides the frequencies of responses to the items on the 2 surveys. The rates of selecting different response options were similar, except that the most extreme positive response was more common for the 2 office staff items on the EZ survey than the CG-CAHPS 3.1 survey.

### Means, Standard Deviations, and Internal Consistency Reliability of Composites

Table 2 shows mean scores for the report composites, which range from 1 to 4, with higher scores representing a better patient experience. The global doctor rating ranges from 0 to 10, with 10 being the best possible score. Internal consistency reliabilities ranged from 0.80 (coordination) to 0.88 (office staff) for the EZ multi-item composites and 0.65 (coordination) to 0.92 (communication) for CG-CAHPS 3.1.

**Table 2.** Means (SDs) and Internal Consistency Reliability (Alpha) Coefficients for EZ and Standard CG-CAHPS 3.1 Survey.

	EZ	CG 3.1
Timely care	3.06 (0.83)	3.09 (0.91)
	0.81 (alpha)	0.90 (alpha)
Office staff	3.53 (0.73)	3.52 (0.65)
	0.88 (alpha)	0.83 (alpha)
Communication	3.65 (0.62)	3.65 (0.65)
	0.83 (alpha)	0.92 (alpha)
Coordination	3.52 (0.75)	3.22 (0.80)
	0.80 (alpha)	0.65 (alpha)
Global rating*	9.09 (1.64)	8.97 (1.83)

* Alpha is not applicable for a single item.

### Patient-Level Correlations Among Measures

As shown in Table 3, product-moment correlations among the patient experience measures at the patient level ranged from the smallest being between timely care and communication (r's = 0.34 and 0.26, EZ and CG-CAHPS 3.1) to the largest between coordination and communication (r's = 0.82 and 0.61, EZ and CG-CAHPS 3.1).

**Table 3.** Correlations Among Scales and Global Doctor Rating (CG-CAHPS 3.1 Survey is Above and EZ Survey is Below the Diagonal).

	Timely	Communication	Coordination	Office staff	Global rating
Timely	**1**.**00**	0.26	0.36	0.28	0.31
Communication	0.34	**1**.**00**	0.61	0.48	0.73
Coordination	0.40	0.82	**1**.**00**	0.48	0.46
Office staff	0.38	0.53	0.53	**1**.**00**	0.42
Global rating	0.37	0.73	0.77	0.33	**1**.**00**

### Provider-Level Reliability and Doctor-Level Correlations Between EZ and CG-CAHPS 3.1 Measures

Table 4 gives provider-level reliability and number of patients needed for 0.70 reliability. Reliability was higher for the EZ measures: timely care (0.44, n = 19), office staff (0.00, n = ∼), communication (0.47, n = 17), coordination (0.19, n = 65), and global rating of the doctor (0.41, n = 21). Provider-level reliability for the CG-CAHPS 3.1 measures was 0 except for coordination (0.08, n = 207).

**Table 4.** Provider-Level Reliability Estimates.

	CG-CAHPS 3.1 survey		EZ survey	
	Reliability	N for 0.70	Reliability	N for 0.70
*Timely care*	0.00	∞	0.44	19
*Communication*	0.00	∞	0.47	17
*Coordination*	0.08	207	0.19	65
*Office staff*	0.00	∞	0.00	∞
*Global rating*	0.38	31	0.41	21

Reliability = (MS_BMS_-MS_WMS_)/MS_BMS_, where MS_BMS_ is between mean square and MS_WMS_ is within mean square, one-way ANOVA with the provider as the between variable.

Abbreviations: ANOVA, analysis of variance; CAHPS^®^, Consumer Assessment of Healthcare Providers and Systems; CG-CAHPS 3.1, Clinician and Group Survey 3.1.

### Item-Scale Correlations

Item-scale correlations (corrected for item overlap with the scale score) for the CG-CAHPS 3.1 survey are provided in Supplemental Table 2 for the 4 composites. One of the coordination of care items (Q13, How often did this provider seem to know the important information about your medical history?) correlated more strongly with the communication composite than it correlated with the sum of the other 2 items in the coordination composite. Supplemental Table 3 provides item-scale correlations for the EZ survey. Like the results for the CG-CAHPS 3.1 survey, 1 of the coordination of care items (Q15, How often did this doctor seem to know what is important to you about your health?) correlated more strongly with the communication composite than with the sum of the other 2 coordination of care items.

### Factor Analyses

Guttman's weakest lower bound (principal component eigenvalues >1) suggested 3 underlying dimensions, whereas parallel analysis indicated a maximum of 4 factors. The scree plot produced from polychoric correlations suggested 4 factors. A 4-factor PROMAX solution showed that Q13 (GC-CAHPS 3.1) and Q15 (EZ) loaded on the communication factor (factor 1). Factor 2 is timely care, factor 3 is office staff, and factor 4 represents care coordination.

Communication and coordination factors were indistinguishable in a 4-factor categorical confirmatory factor analytic model. A 3-factor model that combined the communication and coordination items into a single factor fits the CG-CAHPS 3.1 survey data reasonably well: comparative fit index = 0.95 and root mean square error of approximation = 0.09. The standardized estimates for the 3-factor model for the CG-CAHPS 3.1 and EZ survey are provided in Supplemental Table 4.

## Discussion

Our randomized study of the CG-CAHPS 3.1 and EZ surveys completed by patients of an urban safety net provider on the West Coast yielded noteworthy information. We found low missingness rates and acceptable internal consistency reliability levels for most multi-item patient experience scales. However, we also observed low survey response rates and provider-level reliability estimates. The response rate was higher, and the missing data and skip pattern errors were lower for CG-CAHPS 3.1 (20%) than for the EZ (16%) survey. However, the EZ survey yielded better provider-level reliability.

We implemented several procedures previously recommended to improve response rates, including sending prenotification letters, personalized letters, and survey packets, using first-class postage, sending a second survey as needed, and providing Spanish and English language surveys. We also offered an incentive to complete the survey. Incentives to complete surveys have been shown in prior research to increase response rates. For example, a randomized study of 2 southern California medical centers found that offering a $5 postpaid incentive led to a 57% response rate compared to 50% among those who did not receive an incentive.^18^ In our study, the response rate did not differ significantly between those offered a $2 versus $5 incentive. Mixed mode administration is 1 recommendation for improving the response rate we did not implement because the EZ survey includes visual presentation differences to enhance the readability of the CG-CAHPS CG 3.1 survey. Multiple modes improve response rates because subgroups differ in the data collection strategies for which they are most likely to respond.^1^

The unexpectedly lower response rate for the EZ survey than the CG-CAHPS 3.1 survey highlights the need for a deeper understanding of patient behavior. Despite similar conditions, such as the number of mailings, preferred language, and type of provider, the EZ survey had a lower response rate. This unexpected finding suggests that factors such as familiarity with the survey envelope and the standard CAHPS questionnaire layout may influence response rates, and further investigation into these factors is warranted.

It may also be valuable to evaluate using tablets at the point of care to administer the CG-CAHPS 3.1 and EZ surveys rather than via mail. A qualitative study in a safety net healthcare setting concluded that tablet administration was preferred over paper administration.^19^ A study of tablet administration of the Child HCAHPS survey at the care site indicated higher response rates via tablet (71%) compared to 16% for mail administration.^20^ However, preserving confidentiality when surveys are conducted at the point of care may be challenging. Additionally, the distribution of tablets or surveys by office staff could be problematic if certain types of patients are skipped, affecting the sample's representativeness.

Some attempts to simplify wording for the EZ survey may have contributed to less favorable results than the CG-CAHPS 3.1 survey. For example, dropping the specific reference to this provider's office and changing from “provider” to “doctor” may have increased variability in how patients interpreted the items. The next step could be to conduct cognitive interviews with the existing items and alternative wording to ensure the items are as clear to respondents as possible.

Item-scale correlations and factor analysis indicated some overlap between patient perceptions of communication and coordination of care items. How often the provider seemed to know the important information about the patient's medical history item was more strongly associated with the communication composite than the other items in the coordination of care composite. Consistent with what we found, this “care coordination” item is included in the 5-item communication composite included in the Primary Care First Patient Experience of Care Survey^21^ (https://pcfpecs.org/General-Information/About-PCF-PECS). Moreover, item response theory analysis of responses from seriously ill patients also supported the inclusion of this item as part of the communication scale.^22^ Furthermore, while the correlation between the CG-CAHPS 3.1 communication and coordination of care composites (r = 0.61) and the exploratory factor analysis suggested a distinction, the categorical factor analytic model indicated that all the communication and coordination items loaded together onto a single factor.

## Limitations

This study has limitations. Our West Coast sample may not be representative of other ambulatory clinics. The response rates of 16% for the EZ survey and 20% for the CG-CAHPS 3.1 survey, while higher than the approximate 10% response rate reported for previous patient experience surveys at this federally qualified health center,^23^ still pose a significant challenge. Low response rates are common for surveys involving patients who receive care from safety net providers. The declining response rates are an increasing challenge for all types of surveys.^24^

## Conclusion

Future work with a larger sample is needed to provide additional information about the relative psychometric properties of the CG-CAHPS 3.1 and EZ surveys. This would be beneficial for assessing the robustness of the current study's results but especially useful for evaluating the reliability of the measures at the individual provider or physician group level. It would also provide additional information about the overlap between self-reported care coordination and communication measures.

## Supplemental Material

sj-docx-1-jpx-10.1177_23743735241297622 - Supplemental material for Does an “EZ” Survey Improve the Data Quality of the Consumer Assessment of Healthcare Providers and Systems (CAHPS^®^) Clinician and Group Survey 3.1? Supplemental material, sj-docx-1-jpx-10.1177_23743735241297622 for Does an “EZ” Survey Improve the Data Quality of the Consumer Assessment of Healthcare Providers and Systems (CAHPS^®^) Clinician and Group Survey 3.1? by Ron D. Hays, PhD, Julie A. Brown, BA, Charleen Mikail, MPH, CHES, and Denise D. Quigley, PhD in Journal of Patient Experience
