# Supplementary material for: Does an “EZ” Survey Improve the Data Quality of the Consumer Assessment of Healthcare Providers and Systems (CAHPS®) Clinician and Group Survey 3.1?
Source: J Patient Exp. 2024 Nov 14;11:23743735241297622. doi: 10.1177/23743735241297622 (PMC11565615; doi:10.1177/23743735241297622)
Supplement: sj-docx-1-jpx-10.1177_23743735241297622 - Supplemental material for Does an “EZ” Survey Improve the Data Quality of the Consumer Assessment of Healthcare Providers and Systems (CAHPS®) Clinician and Group Survey 3.1? [file sj-docx-1-jpx-10.1177_23743735241297622.docx]

**Abstract**

Completing self-administered patient experience surveys is challenging for many patients. We randomized adult patients receiving care from an urban safety net provider to complete the Consumer Assessment of Healthcare Providers and Systems (CAHPS®) Clinician and Group Survey 3.1 (CG-CAHPS 3.1), or an “EZ” survey created using plain language principles. We compared response rates, item missingness, item-scale correlations, and reliability of patient experience scores based on 264 completed surveys (64% female, 66% Hispanic, 33% high school education or less). The CG-CAHPS 3.1 survey response rate was higher (20% versus 16%), and failure to follow skip instructions was more common for the EZ survey. Internal consistency reliability for multi-item scales was similar, but provider-level reliability was higher for the EZ than for the CG-CAHPS 3.1 survey measures. Cognitive interviews with patients are needed to assess whether the wording of the EZ survey is responsible for the lower response rates and skip pattern errors. Future studies are also required to provide additional information about the psychometric properties of the CG-CAHPS 3.1 and EZ surveys.

*Key findings*

1. The Consumer Assessment of Healthcare Providers and Systems (CAHPS®) Clinician and Group Survey 3.1 (CG-CAHPS 3.1) yielded a higher response rate than an EZ survey.
2. The CG-CAHPS 3.1 survey produced fewer skip pattern errors by respondents than the EZ survey.
3. The provider-level reliability of EZ survey measures was higher than that of the CG-CAHPS 3.1 survey.
4. The response rate for those offered a $2 versus a $5 incentive did not differ.

**Introduction**

The Consumer Assessment of Healthcare Providers and Systems (CAHPS®) suite of surveys includes items to evaluate experiences with health plans (e.g., health plan and home and community-based services), providers (e.g., clinician and group, hospice, home health, surgical care), facility-based care (hospital, in-center hemodialysis, nursing home, outpatient, and ambulatory surgery), and condition-specific care (cancer, and mental health). CAHPS survey data are used as a component of provider quality payments, including pay-for reporting, hospital value-based purchasing payments, quality bonus payments for Medicare Advantage Plans, and dialysis center value-based purchasing payments.^1^ CAHPS survey users include patients, families, caregivers, healthcare purchasers, healthcare accreditation organizations, providers, health plans, and improvement collaboratives.^2-3^

Surveys must be accessible to ensure that care experiences reflect a representative sample of the target population. Individuals from underserved groups have been shown to have some difficulty completing CAHPS surveys.^4^ Survey completion barriers must be addressed to ensure generalizable research results.^5^

The CAHPS Clinician and Group Survey 3.1 (CG-CAHPS 3.1) was intended to be readable for those with a 7th-grade education or higher. However, the Flesch-Kincaid reading level estimates^6-7^ exceed the target level for 10 of the 12 CG-CAHPS 3.1 core items (Table 1). Supplemental Figure 1 shows an example of a CG-CAHPS 3.1 item estimated to require more than a high school education. The example item has several words written in the passive voice, and line length determines wrapping to the following line. An alternative to this item was created using methods designed to improve the readability^8^ and comprehensibility of questions in healthcare surveys.^9^

We produced an EZ version of the CG-CAHPS 3.1 survey using plain language principles: *grammatic parsing* and *stanzaic versification* (Table 1). Grammatic parsing identifies conjunctives (connector words within sentences) and parses compound and complex sentences into their grammatical components (phrases and clauses); stanzaic versification converts simple survey sentences into 2 or 3 shorter lines to create a survey akin to a stanza in poetry. Each line of the survey stanza, usually a phrase or clause, represents a single idea. Unlike the unsystematic truncation produced by automatic text wrapping from word processing programs, stanzaic versification breaks are designed to represent a single idea. The alternative “EZ” item (shown in Figure 1) is short, presents a single thought on each line, and reduces the estimated reading level to 3^rd^ grade.

The simplification process included changes that could alter the meaning of the corresponding items. In particular, the CG-CAHPS 3.1 items about timely care and office staff refer to this provider’s office, but the EZ items do not. The CG-CAHPS 3.1 communication and coordination of care items refer to “provider,” whereas the EZ survey refers to “doctor.” Finally, a CG-CAHPS 3.1 survey item refers to prescription medicines, but the corresponding EZ item relates to medicine.

The study aimed to inform possible modifications to patient experience surveys. We compare response rates, item missingness, item distributions, item-scale correlations, and reliability of patient experience scores for the CG-CAHPS 3.1 and EZ surveys.

**Method**

*CG-CAHPS 3.1 Survey*. The 31-item CG-CAHPS 3.1 survey includes 9 “About You” questions. It also has one global rating question: “Using any number from 0 to 10, where 0 is the worst provider possible, what number would you use to rate this provider?” Twelve questions were used to elicit patient reports about healthcare:

*EZ Survey.* The EZ survey included 31 questions, including 9 “About You” questions. One global rating question was included: “Rate the care this doctor gave you in the last 6 months. Pick a number from 0 to 10. The Worst doctor is 0. The Best doctor is 10.” Twelve questions were used to elicit patient reports about healthcare:

**Sampling**

We obtained a list of 3,167 patients who had visited a provider in the last six months at 1 of 17 Federally Qualified Health Center urban clinics in [*details* *removed for masking*]. We drew a random sample of 1,600 patients and randomized 800 to complete the CG-CAHPS 3.1 survey and the other 800 to complete the EZ survey. We followed CAHPS guidelines and did not sample more than one person per household. To see if differing incentives improved response rates, we randomized patients to one of two incentive amounts: $2 and $5. Incentives were post-paid, contingent upon returning a survey. The visit dates used for sampling ranged from 1/16/23 through 4/14/23, and survey completion dates ranged from 8/1/23 through 12/31/23.

Several aspects of our field test procedures associated with increasing responses to mail surveys were implemented:^10-11^

- Mailing a pre-notification letter in advance of the survey with the logo of the healthcare organization,
- Using first-class postage,
- Sending a second survey if there is no response to the first mailing,
- Providing a Spanish-language survey to those with a Spanish-language preference noted in their medical record and
- Offering an incentive for completing the survey.

**Analysis**

First, we compare response rates to the CG-CAHPS 3.1 and EZ surveys. Then, we summarize rates of failure to follow skip patterns. We calculated rates of item missingness, item frequencies and means, standard deviations, and estimated alpha internal consistency reliability coefficients^12^ for the timely care, office staff, communication, care coordination, and communication/coordination composites. We estimated the means and standard deviations (SDs) for the global rating of the doctor item. We computed product-moment correlations among the composites and the global rating item at the patient level. We estimated doctor-level reliability and doctor-level correlations among the patient experience measures. We also estimated item-scale correlations^13^ and examined indicators of the number of underlying factors: Guttman’s^14^ weakest lower bound and the scree test.^15^ Then, we evaluated a categorical confirmatory factor analysis model based on the comparative fit index^16^ and the root mean square error of approximation.^17^

All analyses were conducted using SAS 9.4 (TS Level 1M7) software.

**Results**

*Sample.* There were no significant differences between the randomized groups in the number of mailings (EZ mean = 2.01 vs CG-CAHPS 3.1 mean = 2.00, t = 0.47, p = 0.6418), preferred language (EZ 40% Spanish vs CG-CAHPS 3.1 38% Spanish, χ2 =0.62, df=1, p = 0.4311), or type of provider on the sampled visit (χ2 =2.89, df=2, p = 0.2360).

We received 264 completed surveys (147 CG-CAHPS 3.1 survey and 117 EZ survey), a mean of 198 days after the sampled visit (range of 130-329 days) for a response rate of 18% after excluding the 104 surveys that were ineligible due to being undeliverable. The CG-CAHPS 3.1 survey response rate was significantly higher than the EZ survey (20% versus 16%; χ2 =3.85, df=1, p = 0.0497). Response rates did not differ significantly by $5 vs $2 incentive: EZ survey (t = 0.81, df = 743, p=0.4201) and CG-CAHPS 3.1 survey (t = 0.36, df = 749, p =0.7177).

Most of the sample was female (64%). In addition, 66% were Hispanic, 14% Black, 14% White, and 7% Asian. Thirty-three percent reported a high school education or less. Forty-four percent of the surveys were completed in Spanish. The modal age category was 55-64 (40%), and 77% of the sample was between 45 and 74 years old. Self-rated physical health was 3% *poor*, 27% *fair*, 40% *good*, 15% *very good*, and 15% *excellent*. Self-rated mental health was 3% *poor*, 19% *fair*, 36% *good*, 18% *very good*, and 24% *excellent*.

Thirteen CG-CAHPS 3.1 and 18 EZ survey respondents reported not getting care from the named doctor in the last six months, so the analytic sample was 232 surveys (n =133 CG-CAHPS 3.1 survey and n = 99 EZ survey). The patient sample provided assessments of 16 healthcare providers: seven physicians, five nurse practitioners, and four physician assistants.

Among survey respondents, there were no significant differences between CG-CAHPS 3.1 and EZ groups on the $2 vs $5 incentive (t = 0.76, p =.4488), number of mailings (CG-CAHPS 3.1 mean = 1.51, EZ mean = 1.46, t = 0.56, p =0.5760), days between sampled visit and survey return (CG-CAHPS 3.1 mean = 202, EZ mean = 193, t = 1.73, p = .0844), and preferred language (CG 3.1 Spanish = 40%, EZ Spanish = 45%, χ2 = 0.79, df=1, p =.3739). However, there was a significant difference between CG-CAHPS 3.1 and EZ groups on the wave when the survey was returned (CG 3.1 mean = 1.32, EZ mean = 1.10, t = 3.47, p=.0006).

*Failure to Skip*. The Supplemental Appendix details the percentage of the eligible sample failing to follow each of the eight skip pattern sequences. The overall rate of failing to skip was significantly higher for the EZ (26%) than for the CG-CAHPS 3.1 survey (16%): χ2 = 8.04, df=1, p =.0046 (95% CI around the difference 3% to 17%).

*Item Missing Data Rates*. The average number of items missing for the 24 items asked of everyone was small and not significantly different between the EZ and CG-CAHPS 3.1 surveys (1.04 vs 0.53, t =1.78, df = 1, p =0.0769).

*Item Frequencies*. We forward cleaned data based on screener responses so that questions following a screener that were supposed to be skipped were recoded to missing data. Supplemental Table 1 provides the frequencies of responses to the items on the two surveys. The rates of selecting different response options were similar, except that the most extreme positive response was more common for the two office staff items on the EZ survey than the CG-CAHPS 3.1 survey.

*Means, Standard Deviations, and Internal Consistency Reliability of Composites*. Table 2 shows mean scores for the report composites, which range from 1 to 4, with higher scores representing a better patient experience. The global doctor rating ranges from 0 to 10, with 10 being the best possible score. Internal consistency reliabilities ranged from 0.80 (coordination) to 0.88 (office staff) for the EZ multi-item composites and 0.65 (coordination) to 0.92 (communication) for CG-CAHPS 3.1.

*Patient-level Correlations Among Measures*. As shown in Table 3, product-moment correlations among the patient experience measures at the patient level ranged from the smallest being between timely care and communication (r’s = 0.34 and 0.26, EZ and CG-CAHPS 3.1) to the largest between coordination and communication (r’s = 0.82 and 0.61, EZ and CG-CAHPS 3.1).

*Provider-level Reliability and Doctor-Level Correlations Between EZ and CG-CAHPS 3.1 Measures*. Table 4 gives provider-level reliability and number of patients needed for 0.70 reliability. Reliability was higher for all 6 of the EZ measures: timely care (0.44, n = 19), office staff (0.00, n = ~), communication (0.47, n = 17), coordination (0.19, n = 65), communication-coordination (0.38, n = 24), and global rating of the doctor (0.41, n = 21). Provider-level reliability for the CG-CAHPS 3.1 measures was 0 except for coordination (0.08, n = 207).

*Item-Scale Correlations*. Item-scale correlations (corrected for item overlap with the scale score) for the CG-CAHPS 3.1 survey are provided in Supplemental Table 2 for the four composites. One of the coordination of care items (Q13, how often did this provider seem to know the important information about your medical history?) correlated more strongly with the communication composite than it correlated with the sum of the other two items in the coordination composite. Supplemental Table 3 provides item-scale correlations for the EZ survey. Like the results for the CG-CAHPS 3.1 survey, one of the coordination of care items (Q15, how often did this doctor seem to know what is important to you about your health?) correlated more strongly with the communication composite than with the sum of the other two coordination of care items.

*Factor Analyses*. Guttman’s weakest lower bound (principal component eigenvalues >1) suggested 3 underlying dimensions, whereas parallel analysis indicated a maximum of 4 factors. The scree plot produced from polychoric correlations suggested four factors. A four-factor PROMAX solution showed that Q13 (how often did this provider seem to know the important information about your medical history?) loaded on the communication factor (Factor 1). Factor 2 is timely care, Factor 3 is office staff, and Factor 4 represents care coordination.

Communication and coordination factors were indistinguishable in a 4-factor categorical confirmatory factor analytic model. A 3-factor model that combined the communication and coordination items into a single factor fits the CG-CAHPS 3.1 Survey data reasonably well: comparative fit index = 0.95 and root mean square error of approximation = 0.09. The standardized estimates for the three-factor model for the CG-CAHPS 3.1 and EZ survey are provided in Supplemental Table 4.

**Discussion**

Our randomized study of the CG-CAHPS 3.1 and EZ surveys completed by patients of an urban safety net provider on the West Coast yielded noteworthy information. We found low missingness rates and acceptable reliability levels for most multi-item patient experience scales. However, we also observed low survey response rates and provider-level reliability estimates. The response rate was higher, and the missing data and skip pattern errors were lower for CG-CAHPS 3.1 (20%) than for the EZ (16%) survey. However, the EZ survey yielded better provider-level reliability.

We implemented several procedures previously recommended to improve response rates, including sending pre-notification letters, personalized letters, and survey packets, using first-class postage, sending a second survey as needed, and providing Spanish- and English-language surveys. We also offered an incentive to complete the survey. Incentives to complete surveys have been shown in prior research to increase response rates. For example, a randomized study of two southern California medical centers found that offering a $5 post-paid incentive led to a 57% response rate compared to 50% among those who did not receive an incentive.^18^ In our study, the response rate did not differ significantly between those offered a $2 versus $5 incentive. Mixed mode administration is one recommendation for improving the response rate we did not implement because the EZ survey includes visual presentation differences to enhance the readability of the CG-CAHPS CG 3.1 survey. Multiple modes improve response rates because subgroups differ in the data collection strategies for which they are most likely to respond.^1^

The unexpectedly lower response rate for the EZ survey than the CG-CAHPS 3.1 survey highlights the need for a deeper understanding of patient behavior. Despite similar conditions, such as the number of mailings, preferred language, and type of provider, the EZ survey had a lower response rate. This unexpected finding suggests that factors such as familiarity with the survey envelope and the standard CAHPS questionnaire layout may influence response rates, and further investigation into these factors is warranted.

It may also be valuable to evaluate using tablets at the point of care to administer the CG-CAHPS 3.1 and EZ surveys rather than via mail. A qualitative study in a safety net healthcare setting concluded that tablet administration was preferred over paper administration.^19^ A study of tablet administration of the Child HCAHPS survey at the care site indicated higher response rates via tablet (71%) compared to 16% for mail administration.^20^ However, preserving confidentiality when surveys are conducted at the point of care may be challenging. Additionally, the distribution of tablets or surveys by office staff could be problematic if certain types of patients are skipped, affecting the sample's representativeness.

Some attempts to simplify wording for the EZ survey may have contributed to less favorable results than the CG-CAHPS 3.1 survey. For example, dropping the specific reference to this provider’s office and changing from “provider” to “doctor” may have increased variability in how patients interpreted the items. The next step could be to conduct cognitive interviews with the existing items and alternative wording to ensure the items are as clear to respondents as possible.

Item-scale correlations and factor analysis indicated some overlap between patient perceptions of communication and coordination of care items. How often the provider seemed to know the important information about the patient’s medical history item was more strongly associated with the communication composite than the other items in the coordination of care composite. Consistent with what we found, this “care coordination” item is included in the 5-item communication composite included in the Primary Care First Patient Experience of Care Survey^21^ (<https://pcfpecs.org/General-Information/About-PCF-PECS>). Moreover, item response theory analysis of responses from seriously ill patients also supported the inclusion of this item as part of the communication scale.^22^ Furthermore, while the correlation between the CG-CAHPS 3.1 communication and coordination of care composites (r = 0.61) and the exploratory factor analysis suggested a distinction, the categorical factor analytic model indicated that all the communication and coordination items loaded together onto a single factor.

**Limitations**

This study has limitations. Our West Coast sample may not be representative of

other ambulatory clinics. The response rates of 16% for the EZ survey and 20% for the CG-CAHPS 3.1 survey, while higher than the approximate 10% response rate reported for previous patient experience surveys at this federally qualified health center,^23^ still pose a significant challenge. Low response rates are common for surveys involving patients who receive care from safety net providers. The declining response rates are an increasing challenge for all types of surveys.^24^

**Conclusion**

Future work with a larger sample is needed to provide additional information about the relative psychometric properties of the CG-CAHPS 3.1 and EZ surveys. This would be beneficial for assessing the robustness of the current study's results but especially useful for evaluating the reliability of the measures at the individual provider or physician group level. It would also provide additional information about the overlap between self-reported care coordination and communication measures.

**References**

1. Orr N, Zaslavsky AM, Hays RD, et al. Development, methodology, and adaptation of the Medicare Consumer Assessment of Healthcare Providers and Systems (CAHPS®) patient experience survey, 2007-2019. *Health Serv Outcomes Res Methodol*. 2023;23:1-20. doi.org/10.1007/s10742-022-00277-9

2. Darby C, Hays RD, Kletke P. Development and evaluation of the CAHPS hospital survey. *Health Serv Res*. 2005;40(6 Pt 2):1973-1976. doi:10.1111/j.1475-6773.2005.00490.x

3. Quigley DD, Elliott MN, Qureshi N, Predmore Z, Hays RD. How the CAHPS Clinician and Group patient experience survey data have been used in research: A systematic review. *J Patient Cent Res Rev*. 2024;11(2):88-96. doi: 10.17294/2330-0698.2056

4. Fongwa MN, Cunningham W, Weech-Maldonado R, Gutierrez PR, Hays RD. Comparison of data quality for reports and ratings of ambulatory care by African American and White Medicare managed care enrollees. *J Aging Health*. 2006;18(5):707-721. doi:10.1177/0898264306293264

5. Calvert MJ, Cruz Rivera S, Retzer A, et al. Patient reported outcome assessment must be inclusive and equitable. *Nat Med*. 2022;28(6):1120-1124. doi:10.1038/s41591-022-01781-8

6. Flesch R. A new readability yardstick. *J Appl Psychol*. 1948;32(3):221–233. doi:10.1037/h0057532.

7. Kincaid JP, Fishburne RP, Rodgers RL, Chissom BS. *Derivation of new readability formulas (Automated readability index, fog count, and Flesch reading ease formula) for Navy enlisted personnel*. Chief of Naval Technical Training, Naval Air Station Memphis; 1975.

8. Calderón, J. L., Smith, S., Baker, R. S. FONBAYS: a simple method for enhancing readability of patient information. *Ann Behav Sci Med Educ*. 2007;13(1), 20-24.

9. Paz SH, Jones L, Calderón JL, Hays RD. Readability and comprehension of the Geriatric Depression Scale and PROMIS^®^ Physical Function Items in older African Americans and Latinos. *Patient*. 2017;10(1):117-131. doi:10.1007/s40271-016-0191-y

10. Edwards P, Roberts I, Clarke M, et al. Increasing response rates to postal questionnaires: systematic review. *BMJ*. 2002;324(7347):1183. doi:10.1136/bmj.324.7347.1183

11. Dillman DA, Smyth JD, Christian LM, Dillman DA. *Internet, mail, and mixed-mode surveys: The tailored design method*. Hoboken, N.J.: Wiley & Sons; 2009.

12. Cronbach LJ. Coefficient alpha and the internal structure of tests. Psychometrika. 1951;16:297–334. https://doi.org/10.1007/BF02310555

13. Hays RD, Hayashi T. Beyond internal consistency reliability: Rationale and user's guide for Multitrait Scaling Analysis Program on the microcomputer. *Behavior Research Methods, Instruments & Computers*. 1990;22:167-175.

14. Guttman L. (1940). Multiple rectilinear prediction and the resolution into components. *Psychometrika*;1940;5:75-99. doi.org/10.1007/BF02287866

15. Cattell RB. The scree test for the number of factors. *Multivariate Behav Res*. 1966;1(2):245-276. doi:10.1207/s15327906mbr0102_10

16. Bentler PM. Comparative fit indexes in structural models. *Psychol Bull*. 1990;107(2):238-246. doi:10.1037/0033-2909.107.2.238

17. Steiger JH. Understanding the limitations of global fit assessment in structural equation modeling. *Personality and Individual Differences*. 2007;42(5):893-898. doi.org/10.1016/j.paid.2006.09.017.

18. Brown JA, Serrato CA, Hugh M, et al. Effect of a post-paid incentive on response rates to a web-based survey. *Survey Practice*. 2016;9(1). doi.org/10.29115/SP-2016-0001

19. Tieu L, Hobbs A, Sarkar U, Nacev EC, Lyles CR. Adapting patient experience data collection processes for lower literacy patient populations using tablets at the point of care. *Med Care*. 2019;57(Suppl 6 2):S140-S148. doi:10.1097/MLR.0000000000001030

20. Toomey SL, Elliott MN, Zaslavsky AM, et al. Improving response rates and representation of hard-to-reach groups in family experience surveys. *Acad Pediatr*. 2019;19(4):446-453. doi:10.1016/j.acap.2018.07.007

21. Centers for Medicare & Medicaid Services. Primary care first patient experience of care survey. https://pcfpecs.org/Portals/0/SurveyMaterials/PY2024/PCFEnglishQuestionnaire.pdf.

Published 2021. Accessed September 10, 2024.

22. Hays RD, Walling AM, Sudore RL, Chau A, Wenger NS. Support for use of Consumer Assessment of Healthcare Providers and Systems communication items among seriously ill patients. *J Palliat Med*. 2023;26(9):1234-1239. doi:10.1089/jpm.2022.0572

23. Quigley DD, Predmore Z, Martino S, Qureshi N, Hays RD. Patient comments on the Consumer Assessment of Healthcare Providers and Systems Clinician and Group (CG-CAHPS) Survey reflect improvements in provider behaviors from coaching. *J Healthc Manag*. 2023;68(4):251-267. doi:10.1097/JHM-D-22-00140

24. Godden E, Paseka A, Gnida J, Inguanzo J. The impact of response rate on Hospital Consumer Assessment of Healthcare Providers and System (HCAHPS) dimension scores. *Patient Exp J*. 2019;6(1):105-114. doi: 10.35680/2372-0247.1357

**Table 1: CG-CAHPS 3.1 Survey and EZ Survey Items (Flesch-Kincaid reading level)**

| **CG-CAHPS 3.1 Survey** | **EZ Survey** |
| --- | --- |
| *Timely Care* | *Timely Care* |
| 6. In the last 6 months, when you contacted this provider’s office to get an appointment for care you needed right away, how often did you get an appointment as soon as you needed? (13.7) | 6. How often did you get care as soon as you needed? (2.6) |
| 8. In the last 6 months, when you made an appointment for a check-up or routine care with this provider, how often did you get an appointment as soon as you needed? (12.1) | 8. How often did you get an appointment as soon as you needed? (4.8) |
| 10. In the last 6 months, when you contacted this provider’s office during regular office hours, how often did you get an answer to your medical question that same day? (13.2) | 10. How often did you get answers to your medical questions the same day? (5.8) |
| *Communication* | *Communication* |
| 11. In the last 6 months, how often did this provider explain things in a way that was easy to understand? (8.1) | 13. How often did this doctor explain things in a way you understood? (5.8) |
| 12. In the last 6 months, how often did this provider listen carefully to you? (7.5) | 14. How often did this doctor listen to you carefully? (7.5) |
| 14. In the last 6 months, how often did this provider show respect for what you had to say? (5.8) | 16. How often did this doctor show respect for what you had to say? (4.0) |
| 15. In the last 6 months, how often did this provider spend enough time with you? (5.2) | 17. How often did this doctor spend enough time with you? (3.6) |
| *Coordination of care* | *Coordination of care* |
| 13. In the last 6 months, how often did this provider seem to know the important information about your medical history? (11.6) | 15. How often did this doctor seem to know what is important to you about your health? (6.1) |
| 17. In the last 6 months, when this provider ordered a blood test, x-ray, or other test for you, how often did someone from this provider’s office follow up to give you those results? (13.3) | 19. How often did this doctor explain the test results to you? (4.7) |
| 20. In the last 6 months, how often did you and someone from this provider’s office talk about all the prescription medicines you were taking? (11.4) | 21. How often did this doctor talk about all the medicine you took? (5.8) |
| *Office staff* | *Office staff* |
| 21. In the last 6 months, how often were clerks and receptionists at this provider’s office as helpful as you thought they should be? (9.2) | 11. How often were clerks and receptionists as helpful as they should be in the last 6 months? (6.3) |
| 22. In the last 6 months, how often did clerks and receptionists at this provider’s office treat you with courtesy and respect? (10.0) | 12. How often did clerks and receptionists treat you with respect in the last 6 months? (5.9) |

**Table 2: Means (SDs) and internal consistency reliability (alpha)**

**coefficients for EZ and Standard CG-CAHPS 3.1 Survey**

|  | EZ | CG 3.1 |
| --- | --- | --- |
| Timely Care | 3.06 (0.83) | 3.09 (0.91) |
|  | 0.81 (alpha) | 0.90 (alpha) |
| Office Staff | 3.53 (0.73) | 3.52 (0.65) |
|  | 0.88 (alpha) | 0.83 (alpha) |
| Communication | 3.65 (0.62) | 3.65 (0.65) |
|  | 0.83 (alpha) | 0.92 (alpha) |
| Coordination | 3.52 (0.75) | 3.22 (0.80) |
|  | 0.80 (alpha) | 0.65 (alpha) |
| Global rating | 9.09 (1.64) | 8.97 (1.83) |

**Table 3: Correlations Among Scales and Global Doctor Rating (CG-CAHPS 3.1 survey is above and EZ survey is below the diagonal)**

|  | *Timely* | *Communication* | *Coordination* | *Office Staff* | *Global Rating* |
| --- | --- | --- | --- | --- | --- |
| *Timely* | **1.00** | 0.26 | 0.36 | 0.28 | 0.31 |
| *Communication* | 0.34 | **1.00** | 0.61 | 0.48 | 0.73 |
| *Coordination* | 0.40 | 0.82 | **1.00** | 0.48 | 0.46 |
| *Office Staff* | 0.38 | 0.53 | 0.53 | **1.00** | 0.42 |
| *Global Rating* | 0.37 | 0.73 | 0.77 | 0.33 | **1.00** |

**Table 4: Provider-Level Reliability Estimates**

|  | CG-CAHPS 3.1 survey | | EZ survey | |
| --- | --- | --- | --- | --- |
|  | Reliability | N for 0.70 | Reliability | N for 0.70 |
| *Timely care* | 0.00 | ∞ | 0.44 | 19 |
| *Communication* | 0.00 | ∞ | 0.47 | 17 |
| *Coordination* | 0.08 | 207 | 0.19 | 65 |
| *Office Staff* | 0.00 | ∞ | 0.00 | ∞ |
| *Global Rating* | 0.38 | 31 | 0.41 | 21 |

Reliability = (MS_BMS_-MS_WMS_)/MS_BMS_ Where MS_BMS_ = Within between square MS_WMS_ = Within mean square, one-way ANOVA with the provider as the between variable.

**Supplemental Table 1: Percentage of Sample Selecting Each Response Option for CG-CAHPS 3.1 and EZ Surveys**

| **Items** | **Never** | **Sometimes** | **Usually** | **Always** |
| --- | --- | --- | --- | --- |
| *Timely Care* |  |  |  |  |
| Care as soon as needed | 11 (0) | 19 (21) | 25 (37) | 46 (41) |
| Got appointment as soon as needed | 6 (1) | 13 (21) | 36 (40) | 46 (37) |
| Got answers to medical questions same day | 10 (11) | 19 (25) | 21 (22) | 50 (41) |
| *Communication* |  |  |  |  |
| Provider explain things in a way you understand | 2 (3) | 8 (9) | 33 (31) | 57 (57) |
| Provider listen to you carefully | 2 (3) | 6 (7) | 25 (13) | 68 (77) |
| Provider show respect for what you had to say | 3 (1) | 6 (5) | 11 (19) | 80 (75) |
| Provider spend enough time with you | 4 (4) | 5 (6) | 14 (11) | 77 (78) |
| *Coordination* |  |  |  |  |
| Provider know what is important about your health | 2 (2) | 2 (2) | 12 (12) | 83 (83) |
| Provider explain the test results to you | 5 (2) | 6 (9) | 21 (23) | 68 (65) |
| Provider talk about all medicine you take | 4 (2) | 7 (7) | 22 (18) | 67 (73) |
| *Office Staff* |  |  |  |  |
| Clerks and receptionists helpful | 18 (5) | 13 (10) | 12 (13) | 58 (72) |
| Clerks and receptionists treat you with respect | 15 (9) | 15 (11) | 25 (11) | 46 (69) |

Percentages for the CG-CAHPS 3.1 survey are followed by the EZ survey percentages (within parentheses).

**Supplemental Table 2: Item-Scale Correlation Matrix for CG-CAHPS 3.1 Survey (n = 131)**

| Item | Timely Care | Communication | Coordination of Care | Office Staff |
| --- | --- | --- | --- | --- |
| Q6 | 0.82* | 0.30 | 0.32 | 0.35 |
| Q8 | 0.84* | 0.26 | 0.37 | 0.39 |
| Q10 | 0.71* | 0.25 | 0.31 | 0.39 |
| Q11 | 0.26 | 0.88* | 0.59 | 0.53 |
| Q12 | 0.26 | 0.88* | 0.61 | 0.54 |
| Q14 | 0.23 | 0.76* | 0.49 | 0.42 |
| Q15 | 0.28 | 0.75* | 0.46 | 0.39 |
| Q13 | 0.30 | **0.82** | 0.42* | 0.50 |
| Q17 | 0.29 | 0.35 | 0.59* | 0.40 |
| Q20 | 0.27 | 0.35 | 0.57* | 0.36 |
| Q21 | 0.39 | 0.42 | 0.50 | 0.70* |
| Q22 | 0.34 | 0.54 | 0.46 | 0.70* |

Note: Asterisks indicate item-scale correlations for the hypothesized scale (corrected for item overlap with the scale total). The bolded cell indicates that the item (“How often did this provider seem to know the important information about your medical history”) correlated more highly with the communication scale than the coordination of care scale it was hypothesized to represent.

**Supplemental Table 3: Item-Scale Correlation Matrix for EZ Survey (n = 98)**

| Item | Timely Care | Communication | Coordination of Care | Office Staff |
| --- | --- | --- | --- | --- |
| Q6 | 0.80* | 0.38 | 0.42 | 0.30 |
| Q8 | 0.91* | 0.30 | 0.38 | 0.36 |
| Q10 | 0.78* | 0.38 | 0.44 | 0.40 |
| Q13 | 0.43 | 0.82* | 0.72 | 0.53 |
| Q14 | 0.32 | 0.86* | 0.77 | 0.43 |
| Q16 | 0.33 | 0.84* | 0.74 | 0.52 |
| Q175 | 0.32 | 0.76* | 0.77 | 0.50 |
| Q153 | 0.49 | **0.90** | 0.70* | 0.51 |
| Q19 | 0.42 | 0.71 | 0.85* | 0.46 |
| Q21 | 0.32 | 0.67 | 0.77* | 0.48 |
| Q11 | 0.43 | 0.49 | 0.49 | 0.79* |
| Q12 | 0.29 | 0.54 | 0.53 | 0.79* |

Note: Asterisks indicate item-scale correlations for the hypothesized scale (corrected for item overlap with the scale total). The bolded cell indicates that the item (“How often did this provider seem to know the important information about your medical history”) correlated more highly with the communication scale than the coordination of care scale it was hypothesized to represent.

**Supplemental Table 4: Standardized Factor Loadings for 3-Factor Categorical Factor Analytic Model for the CG-CAHPS 3.1 and EZ Surveys**

| **Item** | **Timely** | **Communication** | **Office** |
| --- | --- | --- | --- |
| Q6 (Q6) Care as soon as needed | 0.92 (0.80) |  |  |
| Q8 (Q8) Got an appointment as soon as needed | 0.95 (1.00) |  |  |
| Q10 (Q10) Got answers to questions same day | 0.75 (0.68) |  |  |
| Q11 (Q13) Explained things in a way you understand |  | 0.94 (0.91) |  |
| Q12 (Q14) Listen to you carefully |  | 0.94 (0.87) |  |
| Q14 (Q16) Show respect for what you had to say |  | 0.80 (0.92) |  |
| Q15 (Q17) Spent enough time with you |  | 0.77 (0.84) |  |
| Q13 (Q15) Know what is important about your health |  | 0.87 (0.79) |  |
| Q17 (Q19) Get you the test results |  | 0.32 (0.74) |  |
| Q20 (Q21) Talk about the medicine you take |  | 0.56 (0.79) |  |
| Q21 (Q11) Staff helpful |  |  | 0.82 (0.84) |
| Q22 (Q12) Staff courtesy and respect |  |  | 0.88 (0.92) |

Loadings for the CG-CAHPS 3.1 survey are followed by those for the EZ survey (within parentheses).

**Supplemental Figure 1: Comparison of CG-CAHPS 3.1 and Corresponding EZ Item**


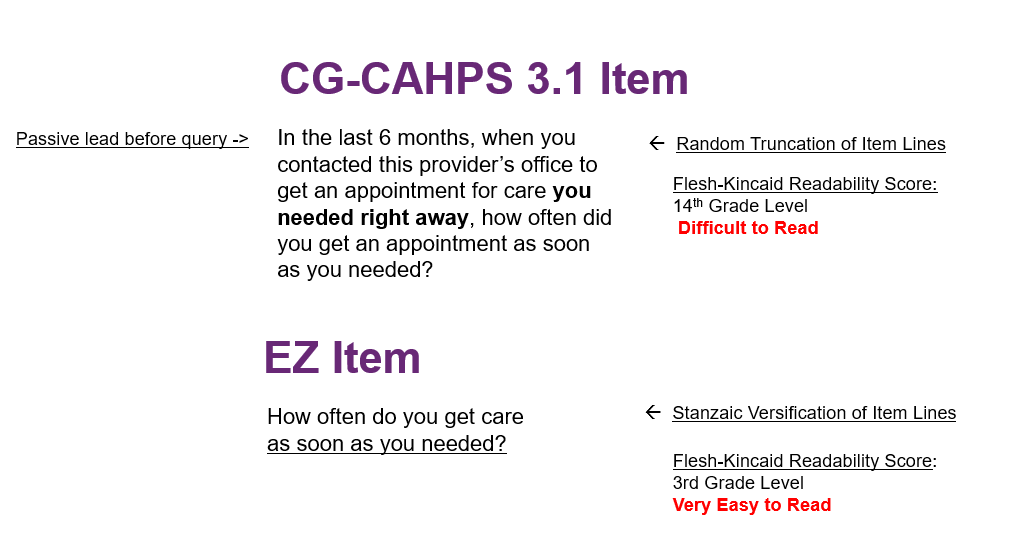


**Supplemental Appendix: Failure to Skip**

The percentage of the eligible sample failing to follow each of the eight skip pattern sequences is below. For example, among the eight people who reported that they did not get care from the doctor named at the beginning of the CG3.1 survey, 12% answered at least one of the questions they were instructed to skip. In comparison, among the 12 people who reported that they did not get care from the doctor named at the beginning of the EZ survey, 33% answered at least one question they were instructed to skip.

**Percentage of Sample Failing to Skip on CG-CAHPS 3.1 and EZ Surveys**

| *Skip #* | *CG-CAHPS 3.1* | *EZ* |
| --- | --- | --- |
| 1 | 12% (n =8) | 33% (n = 12) |
| 2 | 38% (n = 8) | 83% (n = 6) |
| 3 | 25% (n = 44) | 39% (n = 28) |
| 4 | 28% (n = 29) | 55% (n = 11) |
| 5 | 25% (n = 55) | 44% (n = 39) |
| 6 | 8% (n = 26) | 55% (n = 11) |
| 7 | 36% (n = 11) | 27% (n = 15) |
| 8 | 5% (n = 132) | 4% (n = 99) |

CG 3.1 = Consumer Assessment of Healthcare Providers and Systems (CAHPS®) Clinician and Group Survey 3.1; EZ = Parallel survey designed to be easier to read. Numbers within parentheses represent the number of people who were supposed to skip subsequent questions.

**CG-CAHPS 3.1**

Question 1. Among the eight people who reported that they did not get care from the doctor named at the beginning of the questionnaire, 12% answered 3 of the 21 questions they were supposed to skip.

Question 4. Among the eight people who reported not receiving care in the last six months from the doctor named at the beginning of the questionnaire, 38% answered all the questions they were supposed to skip.

Question 5. Among the 44 people who did not contact this doctor’s office to get care they needed right away, 25% answered the next question that they were supposed to skip.

Question 7. Among the 29 people who made an appointment for routine care, 28% answered the next question that they were supposed to skip.

Question 9. Among the 55 people who did not contact the doctor with a medical question in the last six months, 25% answered the next question they were supposed to skip.

Question 16. Among the 26 people for whom the doctor did not order any test in the last six months, 8% answered the next question that they were supposed to skip.

Question 19. Among the 11 people who did not take any prescription medicine in the last six months, 36% answered the next question that they were supposed to skip.

Question 30. Among the 132 people who did not have someone help them complete the survey, 5% answered the next question that they were supposed to skip.

**EZ**

Question 1. Among the 12 people who reported that they did not get care from the doctor named at the beginning of the questionnaire, 33% answered at least 1 of the 21 questions they were instructed to skip: 3 answered 1, and 1 answered most of them.

Question 4. Among the six people who reported not receiving care in the last six months from the doctor named at the beginning of the questionnaire, 83% answered at least 1 of the 18 questions they were instructed to skip: 1 answered 8, and 4 answered all.

Question 5. Among the 28 people who did not contact this doctor’s office to get the care they needed right away, 39% answered the next question that they were supposed to skip.

Question 7. Among the 11 people who made an appointment for routine care, 55% answered the next question that they were instructed to skip.

Question 9. Among the 39 people who did not contact the doctor with a medical question in the last six months, 44% answered the next question they were instructed to skip.

Question 18. Among the 11 people for whom the doctor did not order any test in the last six months, 55% answered the next question that they were instructed to skip.

Question 20. Among the 15 people who did not take prescription medicine in the last six months, 27% answered the next question that they should skip.

Question 30. Among the 99 people who did not have someone to help them complete the survey, 4% answered the next question that they were instructed to skip.
